# Maternal Tobacco Use During Pregnancy and Child Neurocognitive Development

**DOI:** 10.1001/jamanetworkopen.2023.55952

**Published:** 2024-02-13

**Authors:** Troy B. Puga, Hongying Daisy Dai, Yingying Wang, Elijah Theye

**Affiliations:** 1 College of Public Health, University of Nebraska Medical Center, Omaha; 2 College of Osteopathic Medicine, Kansas City University, Kansas City, Missouri; 3 Neuroimaging for Language, Literacy & Learning Laboratory, University of Nebraska at Lincoln, Lincoln

## Abstract

**Importance:**

Maternal tobacco use during pregnancy (MTDP) persists across the globe. Longitudinal assessment of the association of MTDP with neurocognitive development of offspring at late childhood is limited.

**Objectives:**

To examine whether MTDP is associated with child neurocognitive development at ages 9 to 12 years.

**Design, Setting, and Participants:**

This cohort study included children aged 9 and 10 years at wave 1 (October 2016 to October 2018) and aged 11 to 12 years at a 2-year follow-up (wave 2, August 2018 to January 2021) across 21 US sites in the Adolescent Brain Cognitive Development (ABCD) Study. Data were analyzed from June 2022 to December 2023.

**Exposure:**

MTDP.

**Main Outcomes and Measures:**

Outcomes of interest were neurocognition, measured by the National Institutes of Health (NIH) Toolbox Cognition Battery, and morphometric brain measures through the region of interest (ROI) analysis from structural magnetic resonance imaging (sMRI).

**Results:**

Among 11 448 children at wave 1 (mean [SD] age, 9.9 [0.6] years; 5990 [52.3%] male), 1607 children were identified with MTDP. In the NIH Toolbox Cognition Battery, children with MTDP (vs no MTDP) exhibited lower scores on the oral reading recognition (mean [SE] *B* = −1.2 [0.2]; *P* < .001), picture sequence memory (mean [SE] *B* = −2.3 [0.6]; *P* < .001), and picture vocabulary (mean [SE] *B* = −1.2 [0.3]; *P* < .001) tests and the crystallized cognition composite score (mean [SE] *B* = −1.3 [0.3]; *P* < .001) at wave 1. These differential patterns persisted at wave 2. In sMRI, children with MTDP (vs no MTDP) had smaller cortical areas in precentral (mean [SE] *B* = −104.2 [30.4] mm^2^; *P* = .001), inferior parietal (mean [SE] *B* = −153.9 [43.4] mm^2^; *P* < .001), and entorhinal (mean [SE] *B* = −25.1 [5.8] mm^2^; *P* < .001) regions and lower cortical volumes in precentral (mean [SE] *B* = −474.4 [98.2] mm^3^; *P* < .001), inferior parietal (mean [SE] *B* = −523.7 [136.7] mm^3^; *P* < .001), entorhinal (mean [SE] *B* = −94.1 [24.5] mm^3^; *P* < .001), and parahippocampal (mean [SE] *B* = −82.6 [18.7] mm^3^; *P* < .001) regions at wave 1. Distinct cortical volume patterns continued to be significant at wave 2. Frontal, parietal, and temporal lobes exhibited differential ROI, while there were no notable distinctions in the occipital lobe and insula cortex.

**Conclusions and Relevance:**

In this cohort study, MTDP was associated with enduring deficits in childhood neurocognition. Continued research on the association of MTDP with cognitive performance and brain structure related to language processing skills and episodic memory is needed.

## Key Points

**Question:**

Is maternal tobacco usage during pregnancy (MTDP) associated with longitudinal neurocognitive developmental outcomes in offspring in late childhood (ages 9-12 years)?

**Findings:**

In this cohort study including 11 448 children, MTDP, compared with no MTDP, was associated with smaller cortical areas and volumes on structural magnetic resonance imaging among children aged 9 to 12 years, with exposed children exhibiting lower scores on the oral reading recognition, picture vocabulary, and crystallized cognition composite score.

**Meaning:**

These findings suggest that MTDP was associated with decreased language and memory development in children, which may result in long-term consequences on their education and overall growth.

## Teaser

This cohort study examines whether maternal tobacco use during pregnancy is associated with neurocognitive development at ages 9 to 12 years among participants in the Adolescent Brain Cognitive Development Study.

## Introduction

Maternal tobacco use during pregnancy (MTDP) has been shown to have negative consequences on child growth and development. MTDP is a preventable cause of morbidity and mortality to both mother and child.^1,2,3^ Tobacco use during pregnancy remains a global health challenge, with some countries having a prevalence of 30% or higher.^1,4^ Rates of tobacco use tend to be higher in developing countries; however, developed countries have significant levels of tobacco use during pregnancy.^1,4^ Electronic cigarettes (e-cigarettes) and smokeless tobacco, which are marketed as safer alternatives to combustible cigarettes, have further compounded the problem.^1,5,6,7^ These products have shifted harm perceptions in ways that could be detrimental to the growth and development of children.^1,5,6,7^

Childhood is a critical development period for the brain. During early childhood and adolescence, the brain develops by increasing its structural size, changing its gray and white matter composition, and refining synaptic communication.^8,9,10^ This developmental process during early childhood and adolescence is crucial for the educational and social development of children.^8,9,10^ A slowed rate of brain development could predispose children to struggles in academic and social achievement. Nicotine exposure affects nicotinic acetylcholine receptors in the brain by upregulating their expression. Furthermore, it could inhibit DNA synthesis, disrupt brain cell growth, and dysregulate neurotransmitter systems.^11^ Children who initiate tobacco use demonstrate blunted longitudinal neurocognitive development.^12^ These children also have blunted language abilities and damage to language processing structures.^12^ Language perception and processing skills are necessary for progression of a child’s education and reading abilities.^13^

MTDP can affect childhood cognition by impairing functional connectivity between brain regions during auditory processing.^11^ A 2007 study by Julvez et al^14^ examined the associations of MTDP with childhood neurocognition up to age 4 years and found, with the use of the McCarthy Scales of Children’s Abilities, that children with MTDP had decreased global cognitive scores, including decreases in their verbal scores, executive function scores, and working memory scores.^14^ An additional study by Key et al^15^ found that newborn infants whose mothers smoked during pregnancy had a slowed speech processing ability, and another study by Bublitz and Stroud^11^ found that children with MTDP had decreased cerebellum volume and lack of coordination between different brain regions during information and auditory processing. However, limited research has examined the longitudinal associations of MTDP with the neurocognitive maturation of children during late childhood development (ie, ages 9-12 years), which marks key components of neurocognitive development.^16^ To fill this gap, the objective of this study is to investigate whether MTDP is associated with childhood neurocognitive development regarding childhood cognition at baseline and a 2-year follow-up and determine whether there are any longitudinal associations of MTDP use regarding morphometric brain structure. Based on the prior evidence of MTDP, we hypothesize that there will be longitudinal neurocognitive developmental outcomes for children with MTDP.

## Methods

This cohort study was granted an exemption for the secondary analysis of deidentified data from the Adolescent Brain Cognitive Development (ABCD) study by the institutional review board of the University of Nebraska Medical Center. All participants provided written informed consent or assent to participate in the ABCD study. This study adheres to the Strengthening the Reporting of Observational Studies in Epidemiology (STROBE) reporting guideline.

### Participants and Data Collection

This study conducted analysis using data from the ABCD 4.0, which come from the National Data Archive. The ABCD data are derived from a large-sample cohort study, and children aged 9 and 10 years were enrolled in the study between October 2016 and October 2018 at 21 sites across the US.^17^ The University of California, San Diego, institutional review board approved the ABCD study. Participants were recruited through the ABCD school selection probability sample based on a variety of factors, such as socioeconomic status, race and ethnicity, and sex assigned at birth.^18^ Parents or guardians and children were informed of the study and potential risks. Participants and a parent or guardian underwent a complete assessment, including interviews, surveys, neurocognitive testing, and neuroimaging.^18,19,20^ Follow-up (wave 2) cognitive performance and neuroimaging testing was completed during August 2018 to January 2021.

### Study Measures

#### MTDP

Prenatal exposure of medications, drugs, alcohol, and tobacco were obtained through clinical assessment and parental survey of medical history. MTDP was assessed before and after knowing of pregnancy. Typical tobacco products included cigarettes, e-cigarettes, smokeless tobacco, cigars, and hookah. Parents with a yes response to using tobacco before or after knowing about the pregnancy were classified into the MTDP group, with the remainder serving as the control group of no exposure.

#### Neurocognition

Neurocognition was measured using the National Institutes of Health (NIH) Toolbox Cognition Battery, composing 7 tasks measuring neurocognitive performance. The NIH Toolbox Cognition Battery was administered to children at waves 1 and 2. Components of the battery include oral reading recognition, dimensional change card sort, list sorting working memory, flanker inhibitory control and attention, picture sequence memory, picture vocabulary, and pattern comparison processing speed tests. Performance on these components leads to composite cognitive scores for crystallized cognition, fluid cognition, and total cognition. The oral reading recognition and picture vocabulary tests are measures of language skills, and the picture sequence memory test is a measure of episodic memory.^21^ The flanker inhibitory control and attention test and the dimensional change card sort test are measures of executive function. The pattern comparison processing speed tests processing speed, and the list sorting working memory tests working memory.^21^ The crystallized cognition composite score, a measure of previously learned knowledge and skills, is most influenced during childhood and difficult to improve in adulthood.^22^

#### Neuroimaging

Magnetic resonance imaging (MRI) was undertaken at both waves. Methods for MRI were standardized across all ABCD study sites. The ABCD imaging study protocol was designed through a pilot study and uses a 3 Tesla MRI magnet across all sites.^23^ Participants underwent desensitization and simulation to control motion during the scan.^23^ During the scan, structural MRIs were collected using prospective motion correction software.^23^ Image reconstruction was undertaken using FreeSurfer version 5.3.0 (FreeSurfer). MRI quality control was undertaken and standardized to exclude participants with poor neuroimaging to remove potential study confounders. Measures on 34 cortical measures of surface areas and volumes from the Desikan-Killiany-Tourville atlas^24^ were selected for this study due to their ability to demonstrate developmental correlates.^25^

#### Sociodemographic Characteristics

Sociodemographic characteristics measured at wave 1 included age, sex assigned at birth, race and ethnicity, family income, parental education level, premature status, and family difficulties within the past calendar year. Self-reported race and ethnicity were included in analyses because they are considered social constructs rather than genetic or biological classifications, and individuals identified as Asian, Black, Hispanic, White, or other, including American Indian or Alaska Native, Native Hawaiian or Other Pacific Islander, other race or ethnicity, or multiple races.

#### Additional Factors

Potential confounders associated with neurocognition were controlled. We assessed use of alcohol, marijuana, or other illicit drug products, as self-reported by children. Illicit drugs included cocaine, methamphetamine, 3,4-methylenedioxymethamphetamine (MDMA), inhalants, stimulants, anabolic steroids, sedatives, opioids, or over-the-counter cold medicine. Puberty was assessed using the puberty development scale, which includes questions regarding development levels of height, body hair, skin, and voice and facial hair (males) or thelarche and menarche (females). Scores range from 1 to 4, with higher scores indicated a more advanced level of pubertal development. Parent monitoring was assessed using a scale composed of 5 questions about perceived parental monitoring and supervision in the child survey. Scores range from 1 to 5, with higher scores indicated higher levels of parental monitoring.^26^ The school risk environment survey asked 6 questions about school and environmental risk.^27^ Scores range from 1 to 25, with higher scores indicated a better school environment.

### Inclusion and Exclusion Criteria

After removal of participants with missing MTDP or cognition measures, the analytical sample of NIH Toolbox Cognitive Assessment included 11 448 participants at wave 1 and 9846 participants at wave 2. For MRI neuroimaging analysis, participants were excluded if they had missing information, poor neuroimaging, and concerning medical factors. The selection criteria were based on the peer-reviewed study protocol,^12^ and inclusion and exclusion criteria are presented in the Figure.

**Figure.**
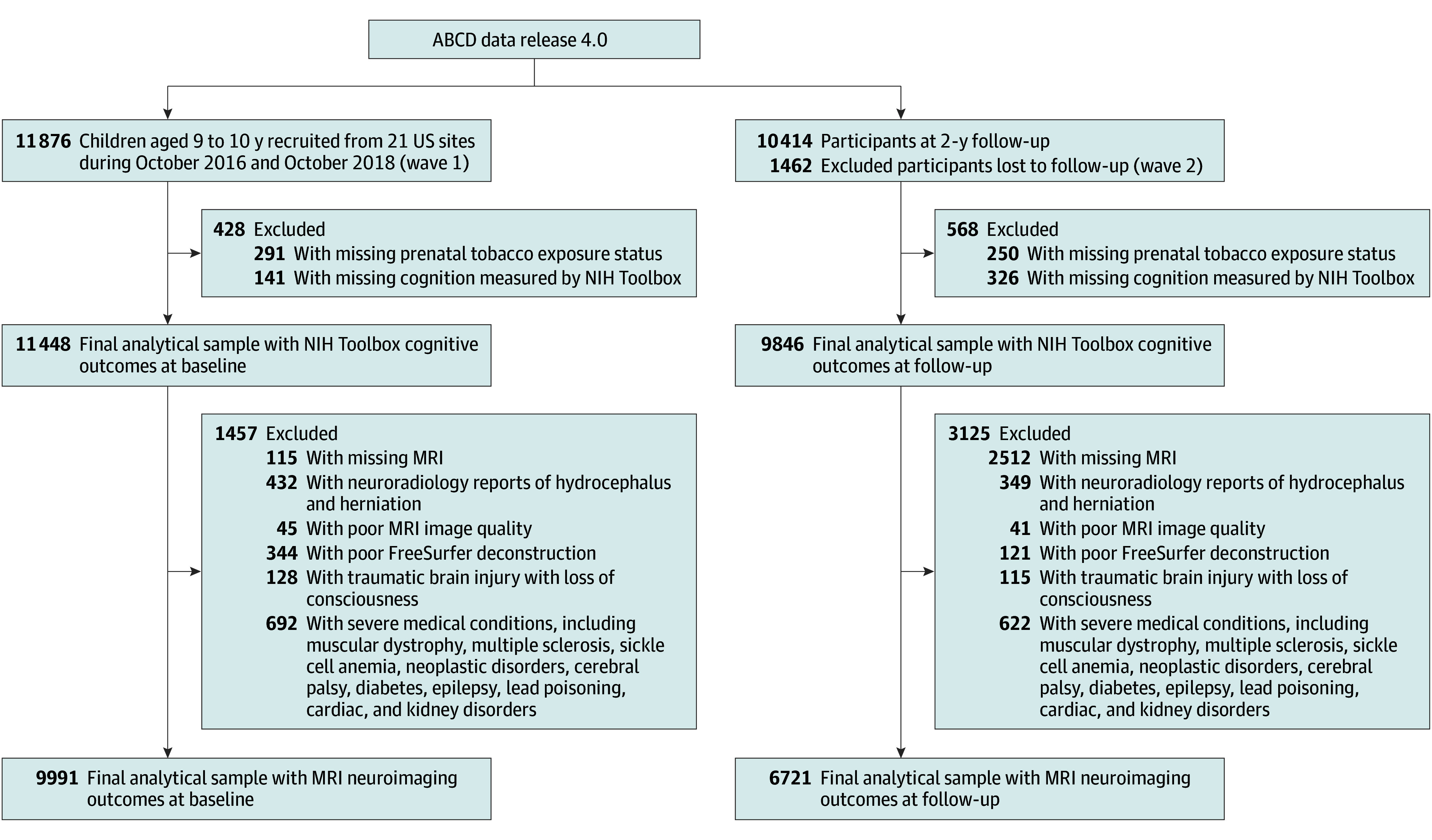
Flowchart of Analytical Sample With Inclusion and Exclusion From the Adolescent Brain Cognitive Development (ABCD) Study^a^ NIH indicates National Institutes of Health. ^a^The selection procedure was based on the protocols in our previous studies and ABCD study magnetic resonance imaging (MRI) quality-control guideline.

### Statistical Analysis

Participant characteristic statistics were compared between the control group and those with prenatal tobacco exposure. To adhere to the ABCD study’s statistical guidelines for population-based analysis, a weighted approach was adopted.^28^ This technique addressed participant clustering across 21 study sites, sample selection biases, and nonresponsiveness within the observational study design. Weighting was accomplished by generating a weight variable through a propensity model incorporating age, sex, and race and ethnicity. Missing data imputation was used to ensure the weighted ABCD data maintained sample demographics consistent with the American Community Survey’s third and fourth grade enrollment statistics for each site.^18,28^ Within the survey analytical procedures, the study sites were treated as clusters. Multivariable regression models were conducted using the Taylor series method to construct variance-covariance matrix for the regression coefficient and estimate sampling errors of estimators, considering the complexities of the study design. The primary analyses assessed the associations between MTDP and measures from the NIH Toolbox Cognition Battery, as well as brain morphometric measures. Models were adjusted for covariates for children, including age, sex assigned at birth, race and ethnicity, use of tobacco, use of other substances, youth pubertal stage, parent monitoring, school environment, and intracranial volume. The domain of structural MRI neuroimaging analysis centered on associations of MTDP with measures of 34 cortical structures (analyzed separately for cortical area and volume) distributed across insula cortex and the frontal, parietal, temporal, and occipital lobes. Multivariable regression models for morphometric measures further included factors like handedness (left vs right) and MRI device manufacturer. Adjusted regression coefficients (*B*) and standard errors (SE) were derived. To ensure the robustness of study findings, propensity score modeling^29^ was further performed in the sensitivity analysis to examine the associations of MTDP and NIH Toolbox measures.

Statistical analyses were conducted using SAS software version 9.4 (SAS Institute). Because of the low incidence of missing data, we used a complete case analysis for our study. To control studywise false discovery rate at *P* = .05, a Benjamini-Hochberg multiple test correction was conducted.^30^ *P* values were 2-sided, and statistical significance was set at *P* = .05. Data were analyzed from June 2022 to December 2023.

## Results

At wave 1, the study population of 11 448 children had a mean (SD) age of 9.9 (0.6) years and included 5990 (52.3%) male participants (Table 1). The population was racially and ethnically diverse, with 221 Asian participants (1.9%), 1668 Black participants (14.6%), 2327 Hispanic participants (20.3%), and 6040 White participants (52.8%). Familial income levels showed 4405 participants (38.5%) had family income greater than $100 000, and 1559 participants (13.6%) reported family difficulties. Among children, 112 children (1.0%) reported tobacco ever use, and 2600 children (22.7%) reported other substance use ever (Table 1). Characteristics data had less than 1% missing rates for all variables at both waves, but 1602 participants were lost to follow up between wave 1 and wave 2 (Table 1).

**Table 1.** Participant Characteristics

Characteristic	Wave 1 (baseline n = 11 448)^a^				Wave 2 (2-y follow-up, n = 9846)^b^			
	Participants, No.	Weighted % (95% CI)		*P* value^d^	Participants, No.	Weighted % (95% CI)		*P* value^d^
		No exposure (n = 9841)	MTDP exposure (n = 1607)^c^			No exposure (n = 8538)	MTDP exposure (n = 1308)^c^	
Age, mean (SD) [95% CI], y	11 448	9.9 (0.6) [8.9-11.1]	9.9 (0.6) [8.9-11.0]	.35	9846	12.0 (0.7) [10.6-13.8]	12.0 (0.7) [10.6-13.8]	.62
Sex								
Male	5990	51.6 (50.3-52.9)	50.8 (47.8-53.8)	.67	5161	51.9 (50.3-53.4)	50.2 (46.7-53.8)	.46
Female	5458	48.4 (47.1-49.7)	49.2 (46.2-52.2)		4685	48.1 (46.6-49.7)	49.8 (46.2-53.3)	
Race and ethnicity								
Asian	221	3.7 (1.6-5.8)	0.8 (0-1.6)	<.001	180	3.5 (1.5-5.5)	0.8 (0-1.7)	<.001
Black	1668	12.5 (7.4-17.6)	16.4 (9.7-23.2)		1288	11.3 (6.4-16.2)	15.0 (8.8-21.3)	
Hispanic	2327	24.9 (11-38.8)	19.4 (10.5-28.2)		1989	24.6 (10.8-38.4)	19.8 (10.6-29.1)	
White	6040	52.8 (39.9-65.7)	53.4 (44.4-62.4)		5373	54.6 (41.5-67.7)	53.9 (45.4-62.3)	
Other^e^	1190	6.1 (4.4-7.8)	10.0 (5.5-14.5)		1016	6.0 (4.2-7.8)	10.5 (5.3-15.6)	
Parental education level								
<High school	645	6.4 (3.3-9.5)	9.1 (5.2-13)	<.001	509	6.0 (2.8-9.2)	8.6 (5-12.3)	<.001
High school diploma or GED	1142	10.3 (8.0-12.5)	19.1 (15.1-23.2)		874	9.2 (7.1-11.4)	17.5 (13.6-21.4)	
Some college or associate degree	2916	25.5 (22.1-28.8)	47.2 (42.4-52.1)		2437	24.8 (21.3-28.4)	47.3 (42.0-52.5)	
Bachelor’s degree	3132	27.7 (23.8-31.6)	15.2 (12.3-18.0)		2802	28.7 (24.8-32.5)	16.6 (13.3-19.9)	
Postgraduate degree	3613	30.2 (25.3-35.0)	9.4 (6.5-12.3)		3224	31.3 (25.9-36.6)	9.9 (7.0-12.8)	
Family income, $								
<25 000	1579	15.3 (11.0-19.7)	26.9 (22.1-31.6)	<.001	1223	13.9 (9.4-18.4)	25.2 (20.4-29.9)	<.001
25 000-49 999	1524	16.5 (13.1-19.9)	26.9 (23.4-30.4)		1313	16.7 (13.2-20.3)	27.1 (23.6-30.5)	
50 000-74 999	1453	15.9 (14.0-17.8)	16.3 (13.1-19.4)		1262	16.1 (14.1-18.2)	17.2 (13.9-20.4)	
75 000-99 999	1509	12.7 (10.6-14.7)	9.6 (7.5-11.6)		1357	13.3 (11.2-15.4)	9.7 (7.2-12.1)	
≥100 000	4405	30.4 (23.3-37.5)	9.9 (7.0-12.7)		3917	31.3 (24.1-38.5)	10.6 (7.3-13.9)	
Do not know or refuse to answer	978	9.2 (7.4-11.0)	10.5 (7.5-13.6)		774	8.6 (6.8-10.4)	10.3 (7.6-13.1)	
Family Difficulty								
No	9889	86.4 (83.9-89)	69.3 (65.3-73.4)	<.001	8596	87.2 (84.6-89.9)	70.7 (65.7-75.6)	<.001
Yes	1559	13.6 (11.0-16.1)	30.7 (26.6-34.7)		1250	12.8 (10.1-15.4)	29.3 (24.4-34.3)	
Premature								
No	9247	81.7 (75.6-87.8)	78.5 (70.8-86.2)	.02	7929	81.3 (74.9-87.7)	78.9 (70.8-86.9)	.10
Yes	2146	18.3 (12.2-24.4)	21.5 (13.8-29.2)		1874	18.7 (12.3-25.1)	21.1 (13.1-29.2)	
Tobacco ever use								
No	11 330	99.1 (98.8-99.4)	97.2 (96.3-98.2)	<.001	7605	99.2 (98.9-99.5)	97.5 (96.3-98.6)	<.001
Yes	112	0.9 (0.6-1.2)	2.8 (1.8-3.7)		2241	0.8 (0.5-1.1)	2.5 (1.4-3.7)	
Other substance ever use								
No	8848	78.3 (74.7-81.9)	76.0 (72.6-79.5)	.24	7605	78.0 (74.2-81.8)	76.5 (73.0-79.9)	.40
Yes	2600	21.7 (18.1-25.3)	24.0 (20.5-27.4)		2241	22.0 (18.2-25.8)	23.5 (20.1-27.0)	
Neighborhood perceptions, mean (SD)								
Child	11 432	4.1 (1.1)	3.7 (1.2)	<.001	9831	4.1 (1.0)	3.8 (1.2)	<.001
Parent	11 408	3.9 (0.9)	3.6 (1.1)	<.001	9812	3.9 (0.9)	3.7 (1.1)	<.001

Abbreviations: GED, general education development; MTDP, maternal tobacco use during pregnancy.

^a^ Missing rate for each variable at wave 1: age, 0%; sex, 0%; race and ethnicity, 0.02%; parental education level, 0%; family income, 0%; family difficulty, 0%; premature, 0.48%; tobacco ever use, 0.05%; other substance ever use, 0%; neighborhood perceptions (child), 0.14%; and neighborhood perceptions (parent), 0.35%.

^b^ Missing rate for each variable at wave 2: age, 0%; sex, 0%; race and ethnicity, 0%; parental education level, 0%; family income, 0%; family difficulty, 0%; premature, 0.44%; tobacco ever use, 0%; other substance ever use, 0%; neighborhood perceptions (child), 0.15%; and neighborhood perceptions (parent), 0.35%.

^c^ Two separate variables were further created to measure MTDP before knowing of pregnancy and after knowing of pregnancy. At wave 1, 995 children (61.9%) were exposed to MTDP before the pregnancy was known and 612 children (38.1%) were exposed to MTDP after the pregnancy was known. At wave 2, 819 children (62.6%) were exposed to MTDP before the pregnancy known, and 489 children (37.4%) were exposed to MTDP after the pregnancy was known. The sample characteristics of MTDP between before and after knowing pregnancy were comparable between these groups, except race and ethnicity and education.

^d^ Rao-Scott χ^2^ tests were performed to compare weighted characteristics of children by MTDP status, accounting for sampling weights and site clustering.

^e^ Individuals identified as American Indian or Alaska Native, Native Hawaiian or other Pacific Islander, other races or multiracial groups.

At wave 1 in the NIH Toolbox Cognition Battery, children with MTDP (vs no exposure) exhibited lower scores on the oral reading recognition (mean [SE] *B* = −1.2 [0.2]; *P* < .001), picture sequence memory (mean [SE] *B* = −2.3 [0.6]; *P* < .001), picture vocabulary tests (mean [SE] *B* = −1.2 [0.3]; *P* < .001), crystallized cognition composite score (mean [SE] *B* = −1.3 [0.3]; *P* < .001), fluid cognition composite score (mean [SE] *B* = −1.8 [0.5]; *P* = .002), and total cognition composite score (mean [SE] *B* = −1.9 [0.4]; *P* < .001) (Table 2). These differential patterns persisted at wave 2 (Table 2). Missing data at wave 1 were low, with less than 1% missing rates for all categories. Wave 2 had more missing data, particularly for the dimensional charge card sort, list sort working memory, and total cognition. This was primarily due to lack of testing in these categories (Table 2). Results from the sensitivity analysis using the propensity score model were consistent with the main analyses (eTable in Supplement 1).

**Table 2.** Longitudinal Comparison of Cognitive Performance Among Children by MTDP^a^

NIH Toolbox Cognition Battery	Wave 1 (baseline)^b^					Wave 2 (2-y follow-up)^c^				
	Mean (SD)		Adjusted *B* (SE)^d^	Standardized *b*	Adjusted P value	Mean (SD)		Adjusted *B* (SE)^d^	Standardized *b*	Adjusted P value
	No exposure (n = 9841)	MTDP exposure (n = 1607)				No exposure (n = 8538)	MTDP exposure (n = 1308)			
Dimensional charge card sort	92.5 (0.3)	91.1 (0.4)	−1.3 (0.3)	−0.05	.001^e^	93.0 (1.4)^f^	96.1 (1.5)^f^	3.1 (2.2)^f^	0.11^f^	.17^f^
Flanker inhibitory control and attention	94.0 (0.3)	93.3 (0.4)	−0.6 (0.3)	−0.03	.03^e^	100.1 (0.3)	99.2 (0.4)	−0.8 (0.4)	−0.04	.07
List sorting working memory	96.5 (0.5)	94.3 (0.6)	−1.8 (0.4)	−0.05	<.001^e^	95.1 (2.2)^f^	93.3 (4.5)^f^	−6.1 (4.9)^f^	−0.12^f^	.22^f^
Oral reading recognition	90.9 (0.2)	89.3 (0.3)	−1.2 (0.2)	−0.07	<.001^e^	95.0 (0.3)	93.3 (0.4)	−1.6 (0.3)	−0.09	<.001^e^
Pattern comparison process speed	88.1 (0.5)	87.3 (0.6)	−0.5 (0.7)	−0.01	.49	103.5 (0.5)	102.6 (0.6)	−0.3 (0.7)	−0.01	.69
Picture sequence memory	103.0 (0.4)	100.8 (0.5)	−2.3 (0.6)	−0.07	<.001^e^	108.9 (0.3)	106.3 (0.8)	−2.3 (0.8)	−0.07	.005^e^
Picture vocabulary tests	84.3 (0.5)	82.7 (0.4)	−1.2 (0.3)	−0.06	<.001^e^	88.8 (0.5)	86.9 (0.5)	−1.5 (0.4)	−0.06	.001^e^
Crystallized cognition composite score	86.3 (0.4)	84.6 (0.4)	−1.3 (0.3)	−0.07	<.001^e^	90.8 (0.4)	89.2 (0.4)	−1.5 (0.3)	−0.08	<.001^e^
Fluid cognition composite score	91.6 (0.5)	89.6 (0.6)	−1.8 (0.5)	−0.06	.002^e^	92.4 (2.1)^f^	91.3 (1.6)^f^	−6.4 (5.4)^f^	−0.18^c^	.25^f^
Total cognition composite score	86.2 (0.5)	84.0 (0.6)	−1.9 (0.4)	−0.08	<.001^e^	86.9 (1.7)^f^	85.4 (1.5)^f^	−6.3 (3.2)^f^	−0.22^f^	.06^f^

Abbreviations: MTDP, maternal tobacco use during pregnancy; NIH, National Institutes of Health.

^a^ Multivariate regression analyses were performed in which dependent variables were cognitive performance scores. Sampling weights and site clustering were incorporated in the survey regression analytical procedures for statistical inference at population level. The independent variable was MTDP (yes vs no).

^b^ Missing rate at wave 1: dimensional charge card sort, 0.10%; flanker inhibitory control and attention, 0.10%; list sorting working memory, 0.46%; oral reading recognition, 0.17%; pattern comparison process speed, 0.24%; picture sequence memory, 0.16%; picture vocabulary tests, 0.05%; crystallized cognition composite score, 0.33%; fluid cognition composite score, 0.79%; and total cognition composite score, 0.83%.

^c^ Missing rate at wave 2: dimensional charge card sort, 99.3%; flanker inhibitory control and attention, 21.4%; list sorting working memory, 99.3%; oral reading recognition, 2.7%; pattern comparison process speed, 21.8%; picture sequence memory, 2.0%; picture vocabulary tests, 2.3%; crystallized cognition composite score, 26.1%; fluid cognition composite score, 99.3%; and total cognition composite score, 99.3%.

^d^ Analysis was adjusted by covariates, including age, sex, race and ethnicity, intracranial volume, pubertal stage, substance ever use, tobacco ever use, parental monitoring, school environment, and study site. Regression coefficients measured the difference in cognitive performance scores by MTDP.

^e^ FDR correction was performed to prevent inflation of type 1 errors.

^f^ Very small sample size of fewer than 100 participants in the no exposure group and fewer than 10 participants in MTDP exposure group. Sensitivity analysis was performed by removing the tests with small sample sizes with consistent results.

In the cortical surface area analysis at wave 1, children with MTDP (vs no MTDP) demonstrated smaller areas in the precentral (mean [SE] *B* = −104.2 [30.4] mm^2^; *P* = .001), inferior parietal (mean [SE] *B* = −153.9 [43.4] mm^2^; *P* < .001), postcentral (*B* = −77.1 [27.9] mm^2^; *P* = .006), and entorhinal (mean [SE] *B* = −25.1 [5.8] mm^2^; *P* < .001) regions. At wave 2, children with MTDP (vs no MTDP) had a smaller area in the precentral region (mean [SE] *B* = −141.2 [38.9]; *P* < .001) (Table 3).

**Table 3.** Region of Interest Analysis of Cortical Area^a^

Region of interest	Wave 1 (baseline, n = 9991)					Wave 2 (2-y follow-up, n = 6721)				
	Cortical area, weighted mean (SE), mm^2^		Adjusted *B *(SE)^b^	Adjusted *P* value	FDR^c^	Cortical area, weighted mean (SE), mm^2^		Adjusted *B *(SE)^b^	Adjusted *P* value	FDR^c^
	No exposure (n = 8634)	MTDP exposure (n = 1357)				No exposure (n = 5812)	MTDP exposure (n = 909)			
**Frontal lobe**										
Superior frontal	15 979.0 (92.0)	15 760.0 (127.4)	−54.2 (49.6)	.28	.47	16 147.0 (87.7)	15 887 (166.7)	−47.6 (60.6)	0.43	.70
Rostral middle	3558.0. (93.1)	13 379.0 (84.1)	−27.8 (45.5)	.54	.72	13 631.0 (85.8)	13 432 (103.4)	−8.3 (58)	0.89	.95
Caudal middle	4929.3 (43.3)	4846.4 (62.2)	−42.7 (24.0)	.08	.21	4986.2 (41.5)	4890.1 (82.1)	−52.5 (30.0)	.08	.24
Pars opercularis	3327.8 (20.4)	3297.2 (26.9)	−1.1 (15.9)	.94	.94	3349.7 (20.1)	3300.6 (25.6)	−5.7 (19.1)	.77	.94
Pars triangularis	3312.2 (17.5)	3291.8 (21.9)	9.2 (15.3)	.55	.72	3321.9 (18.4)	3295.5 (21.8)	32.7 (18.9)	.08	.24
Pars orbitalis	1753.1 (8.5)	1728.2 (8.1)	−10.8 (6.2)	.08	.21	1766.8 (9.8)	1737.6 (8.4)	−3.5 (7.9)	.65	.89
Lateral orbitofrontal	5745.1 (30.9)	5662.3 (36.5)	−8.5 (16.5)	.61	.74	5812.2 (35.3)	5690.4 (48.4)	−30.4 (21.2)	.15	.40
Medial orbitofrontal	4105.7 (21.9)	4042.2 (28.5)	−11.7 (10.5)	.27	.47	4147.0 (24.9)	4057.6 (36.2)	−24.4 (12.8)	.06	.21
Precentral	10 243 (71.4)	10 062 (105.7)	−104.2 (30.4)	.001	.007	10 349 (74.4)	10 127 (140.4)	−141.2 (38.9)	<.001	.01
Paracentral	3049.9 (11.5)	3010.0 (15.5)	−12.1 (10.7)	.26	.47	3058.9 (12.3)	3006.1 (18.9)	−16.4 (13.2)	.21	.45
Frontal pole	677.1 (3.1)	665.6 (2.6)	−3.8 (2.2)	.09	.21	679.5 (3.3)	662.5 (3.4)	−6.9 (2.7)	.009	.14
Rostral anterior	1577.8 (8.2)	1538.9 (8.2)	−5.9 (7.2)	.42	.64	1604.9 (8.5)	1559.1 (12.8)	−6.4 (9.4)	.50	.73
Caudal anterior	1489.8 (8.1)	1465.6 (9.1)	0.8 (8.2)	.92	.94	1521.1 (8.3)	1488.3 (10.3)	0.3 (10.6)	.98	.99
**Parietal lobe**										
Superior parietal	11 930.0 (90.5)	11 779.0 (120.1)	−24.4 (43.2)	.57	.72	11 867.0 (88.4)	11 737.0 (154.4)	15.6 (53.8)	.77	.93
Inferior parietal	11 272.0 (72.1)	10 996.0 (84.7)	−153.9 (43.4)	<.001	.007	11 218.0 (69.4)	10 916.0 (93.1)	−102.6 (53.8)	.06	.21
Supramarginal	8665.0 (70.9)	8475.2 (93.7)	−80.1 (37.9)	.04	.13	8648.9 (64.4)	8469.7 (108.0)	−63.2 (46.5)	.17	.42
Postcentral	8802.5 (72.0)	8643.6 (94.6)	−77.1 (27.9)	.006	.05	8804.4 (73.3)	8645.2 (119.9)	−68.7 (34.6)	.05	.21
Precuneus	8642.4 (31.7)	8508.9 (41.3)	8.7 (29.5)	.77	.87	8588.9 (36.4)	8437.0 (44.9)	25.5 (35.8)	.48	.73
Posterior cingulate	2694.6 (9.6)	2631.1 (16.0)	−26.1 (10.0)	.009	.05	2701.5 (10.8)	2628.1 (16.5)	−26.8 (12.2)	.03	.19
Isthmus cingulate	2161.2 (7.3)	2137.5 (12.9)	−1.9 (8.7)	.83	.91	2163.4 (8.63)	2128.3 (16.2)	−5.4 (10.4)	.61	.85
**Temporal lobe**										
Superior temporal	8647.7 (54.9)	8345.6 (64.2)	−52.6 (25.1)	.04	.13	8481.8 (47.2)	8340.8 (74.6)	−36.3 (31.7)	.25	.51
Inferior temporal	7362.4 (46.2)	7237.1 (68.2)	−25.8 (24.8)	.30	.48	7445.2 (43.4)	7335.7 (87.3)	24.4 (31.0)	.43	.70
Middle temporal	7659.0 (58.6)	7538.5 (81.5)	−18.1 (24.2)	.45	.67	7709.4 (53.5)	7589.5 (97.7)	0.2 (30.6)	.99	.99
Banks of superior temporal sulcus	2174.4 (16.1)	2146.7 (19.8)	−12.3 (9.5)	.20	.39	2168.9 (14.5)	2144.0 (21.7)	−1.6 (11.8)	.89	.95
Fusiform	6646.2 (22.4)	6541.3 (20.1)	−41.4 (19.9)	.04	.13	6702.7 (24.3)	6758.7 (22.3)	−24.7 (23.9)	.30	.57
Transverse temporal	843.8 (3.4)	834.2 (3.2)	−2.6 (3.6)	.48	.67	843.4 (3.5)	830.7 (3.7)	−3.8 (4.4)	.39	.70
Entorhinal	842.3 (5.0)	814.9 (7.2)	−25.1 (5.8)	<.001	<.001	854.2 (4.7)	825.6 (7.1)	−16.8 (6.8)	.01	.14
Temporal pole	997.1 (3.9)	981.6 (4.3)	−6.1 (3.9)	.12	.28	1006.1 (5.0)	990.7 (4.1)	−1.1 (4.6)	.81	.95
Parahippocampal	1317.9 (5.0)	1300.1 (3.8)	−8.7 (4.8)	.07	.21	1338.5 (5.4)	1313.4 (3.8)	−10.4 (6.0)	.09	.24
**Occipital lobe**										
Lateral occipital	11 051.0 (66.0)	10 934 (67.0)	−16.2 (38.7)	.68	.79	11 056 (67.6)	10 922.0 (78.4)	−6.9 (47.4)	.88	.95
Lingual	6723.2 (26.8)	6608.2 (27.0)	−59.8 (26.5)	.02	.12	6761.8 (34.4)	6627.1 (38.7)	−64.4 (31.7)	.04	.21
Cuneus	3386.8 (15.0)	3334.4 (18.0)	−18.9 (13.8)	.17	.36	3396.6 (16.4)	3334.6 (21.7)	−22.5 (17.1)	.19	.43
Pericalcarine	3199.3 (15.3)	3127.3 (16.8)	−48.0 (18.1)	.008	.05	3221.7 (17.7)	3140.9 (25.9)	−52.0 (21.7)	.02	.14
**Insula cortex **										
Insula	4732.7 (18.1)	4681.6 (21.5)	−2.4 (13.3)	.86	.91	4793.4 (20.5)	4727.7 (19.2)	6.0 (16.7)	.72	.94

Abbreviations: FDR, false discovery rate; MTDP, maternal tobacco use during pregnancy.

^a^ Multivariate regression analyses were performed where the dependent variables were cortical areas in 34 regions of interest. The independent variable was MTDP. The analysis was adjusted by covariates, including age, sex, race and ethnicity, intracranial volume, pubertal stage, substance ever use, tobacco ever use, parental monitoring, school environment, handedness, imaging device manufacturer, and study site. Sampling weights were incorporated to remove the sampling bias. No missing data were found among all cortical area measurements.

^b^ Regression coefficients measured the difference in participant structural magnetic resonance imaging variables by MTDP.

^c^ FDR correction was performed across 34 regions to prevent inflation of type I errors.

In cortical volume analysis at wave 1, children with MTDP (vs no MTDP) had lower volume in pars orbitalis (mean [SE] *B* = −67.2 [25.5] mm^3^; *P* = .008), precentral (mean [SE] *B* = −474.4 [98.2] mm^3^; *P* < .001), inferior parietal (mean [SE] *B* = −523.7 [136.7]; *P* < .001), supramarginal (mean [SE] *B* = −330.0 [120.0] mm^3^; *P* = .006), fusiform (mean [SE] *B* = −188.2 [73.5] mm^3^; *P* = .01), entorhinal (mean [SE] *B* = −94.1 [24.5] mm^3^; *P* < .001), and parahippocampal (mean [SE] *B* = −82.6 [18.7] mm^3^; *P* < .001) regions (Table 4). At wave 2, MTDP was associated with lower volumes in the precentral (mean [SE] *B* = −717.3 [120.8] mm^3^; *P* < .001), inferior parietal (mean [SE] *B* = −485.6 [164.3] mm^3^; *P* = .003), entorhinal (mean [SE] *B* = −95.7 [28.2] mm^3^; *P* = .001), and parahippocampal (mean [SE] *B* = −93.9 [23.0] mm^3^; *P* < .001) regions (Table 4).

**Table 4.** Region of Interest Analysis of Cortical Volume^a^

Region of interest		Wave 1 (baseline; n = 9991)										Wave 2 (2-y follow-up; n = 6721)									
		Cortical volume, weighted mean (SE), mm^3^				Adjusted *B *(SE)^b^		Adjusted *P* value		FDR^c^		Cortical volume, weighted mean (SE), mm^3^				Adjusted *B *(SE)^b^		Adjusted *P* value		FDR^c^	
		No exposure (n = 8634)		MTDP exposure (n = 1357)								No exposure (n = 5812)		MTDP exposure (n = 909)							
**Frontal lobe**																				
Superior frontal	57 971.0 (312.2)		57 075.0 (384.3)		−253.6 (168.8)		.13		.28		57 549.0 (321.2)		56 293.0 (479.5)		−381.3 (203.9)		.06		.15	
Rostral middle	42 632.0 (248.7)		41 985.0 (159.3)		−169.2 (143.0)		.24		.42		41 951.0 (268.3)		41 073.0 (232.8)		−296.3 (171.3)		.08		.17	
Caudal middle	16 132.0 (139.0)		15 824.0 (166.0)		−162.6 (81.6)		.05		.14		16 103.0 (130.8)		15 673.0 (193.9)		−249.8 (96.7)		.01		.06	
Pars opercularis	11 037.0 (56.5)		10 976.0 (72.9)		24.4 (53.3)		.65		.75		10 969.0 (61.5)		10 832.0 (65.9)		−20.5 (63.9)		.75		.80	
Pars triangularis	10 826.0 (41.6)		10 795.0 (55.9)		56.8 (53.6)		.29		.49		10 633.0 (55.7)		10 565.0 (62.3)		96.5 (63.9)		.13		.22	
Pars orbitalis	6978.6 (29.7)		6877.6 (26.3)		−67.2 (25.5)		.008		.05		6863.6 (36)		6733.5 (28.5)		−58.4 (30.9)		.06		.15	
Lateral orbitofrontal	18 538.0 (104.1)		18 222.0 (118.9)		−101.3 (54.4)		.06		.16		18 411.0 (117.9)		17 981.0 (160.9)		−160.9 (67.8)		.02		.07	
Medial orbitofrontal	12 698.0 (91.2)		12 521.0 (125.5)		−33.4 (36.9)		.37		.54		12 534.0 (93.7)		12 272.0 (148.8)		−84.9 (43.5)		.05		.14	
Precentral	31 644.0 (146.1)		30 861.0 (167.5)		−474.4 (98.2)		<.001		<.001		31 733.0 (147.2)		30 642.0 (189.9)		−717.3 (120.8)		<.001		<.001	
Paracentral	9386.9 (38.0)		9193.2 (45.3)		−69.4 (35.4)		.05		.14		9218.6 (38.5)		8963.0 (46.7)		−109.4 (43.0)		.01		.06	
Frontal pole	2995.8 (20.9)		2961.9 (17.3)		−8.7 (12.0)		.47		.63		2914.7 (21.1)		2859.3 (15.1)		−20.4 (13.8)		.14		.22	
Rostral anterior	5542.3 (36.9)		5433.2 (32)		−16.5 (27.2)		.54		.68		5541.0 (35.3)		5402.4 (41.2)		−16.5 (33.7)		.63		.71	
Caudal anterior	4631.9 (27.9)		4561.0 (29.2)		4.3 (29.5)		.88		.91		4637.5 (27.0)		4539.9 (34.6)		9.0 (37.2)		.81		.81	
**Parietal lobe**																				
Superior parietal	34 319.0 (193.1)		33 826.0 (247.3)		−78.6 (126.0)		.53		.68		33 262.0 (210.3)		32 703.0 (328.8)		−96.6 (150.4)		.52		.66	
Inferior parietal	35 651.0 (143.6)		34 750.0 (189.9)		−523.7 (136.7)		<.001		.001		34 690.0 (187.9)		33 627.0 (202.9)		−485.6 (164.3)		.003		.03	
Supramarginal	27 899.0 (113.2)		27 188.0 (117.7)		−330.0 (120.0)		.006		.04		27 348.0 (138.2)		26 570.0 (149.6)		−364.0 (148.1)		.01		.06	
Postcentral	23 544.0 (105.5)		23 003.0 (148.8)		−224.5 (88.3)		.01		.05		23 034.0 (109.1)		22 367.0 (174.0)		−266.6 (109)		.01		.06	
Precuneus	25 470.0 (114.3)		25 112.0 (142.9)		48.7 (88.8)		.58		.71		24 670.0 (128.9)		24 257.0 (148)		79.3 (106.1)		.46		.62	
Posterior cingulate	8042.6 (28.8)		7857.1 (54.6)		−72.2 (31.3)		.02		.08		7900.3 (32.7)		7687.8 (52.0)		−59.1 (36.9)		.11		.21	
Isthmus cingulate	6290.3 (25.1)		6229.6 (46.2)		2.2 (27.1)		.94		.94		6153.4 (31.0)		6055.3 (56.4)		−13.4 (31.6)		.67		.74	
**Temporal lobe**																				
Superior temporal	30 487.0 (141.6)		30 077.0 (108.1)		−171.0 (94.0)		.07		.17		30 118.0 (158.0)		29 577.0 (143.1)		−242.5 (114.9)		.04		.11	
Inferior temporal	26 668.0 (136.9)		26 131.0 (217.9)		−163.6 (95.5)		.09		.20		26 536.0 (144.6)		25 955.0 (274)		−83.2 (113.7)		.46		.62	
Middle temporal	29 699.0 (136)		29 181.0 (161.4)		−94.7 (92.2)		.31		.49		29 456.0 (161.8)		28 754.0 (199.2)		−245.6 (113.2)		.03		.10	
Banks of superior temporal sulcus	5886.1 (33.5)		5776.2 (40.9)		−60.9 (29.2)		.04		.13		5775.1 (32.4)		5658.2 (43.0)		−52.5 (35.7)		.14		.22	
Fusiform	22 206.0 (92.0)		21 822.0 (92.9)		−188.2 (73.5)		.01		.05		22 063.0 (98)		21 585 (101.1)		−156.8 (85.6)		.07		.15	
Transverse temporal	2635.0 (14.1)		2605.4 (16.8)		−3.9 (13.7)		.78		.83		2595.7 (16.5)		2559.7 (16.6)		−4.2 (15.9)		.79		.81	
Entorhinal	3729.9 (23.0)		3612.0 (24.7)		−94.1 (24.5)		<.001		.001		3784.9 (20.9)		3633.6 (33.7)		−95.7 (28.2)		.001		.008	
Temporal pole	5462.9 (26.8)		5391.0 (27.0)		−35.1 (24.1)		.15		.29		5468.7 (23.9)		5383.9 (21.6)		−42.9 (28.8)		.14		.22	
Parahippocampal	4374.4 (20.6)		4272.3 (20.9)		−82.6 (18.7)		<.001		<.001		4385.4 (23.1)		4250.5 (25.2)		−93.9 (23.0)		<.001		.001	
**Occipital lobe**																				
Lateral occipital	29 159.0 (177.6)		28 821.0 (175.2)		47.3 (112.2)		.67		.75		28 594.0 (199.5)		28 065.0 (183.0)		−93 (128.9)		.47		.62	
Lingual	16 019.0 (91.1)		15 807.0 (103.7)		−53.1 (72.3)		.46		.63		15 750.0 (98.3)		15 449.0 (116.3)		−121.9 (84.5)		.15		.22	
Cuneus	7908.0 (49.6)		7839.8 (61.1)		39.4 (41.7)		.35		.53		7709.6 (53.1)		7577.5 (70)		−24.5 (48.4)		.61		.71	
Pericalcarine	5157.0 (360)		5060.9 (45.8)		−45.3 (33.9)		.18		.34		5105.9 (42.6)		4978.6 (69.6)		−67.6 (39.3)		.09		.17	
**Insula cortex**																				
Insula	15 283.0 (84.9)		15 152 (100.4)		18.5 (45.3)		.68		.75		15 276.0 (83.9)		15 108.0 (89.7)		29.8 (53.3)		.58		.70	

Abbreviations: FDR, false discovery rate; MTDP, maternal tobacco use during pregnancy.

^a^ Multivariate regression analyses were performed where the dependent variables were cortical volumes in 34 regions of interest. The independent variable was MTDP. The analysis was adjusted by covariates, including age, sex, race/ethnicity, intracranial volume, pubertal stage, substance ever use, tobacco ever use, parental monitoring, school environment, handedness, imaging device manufacturer, and study site. Sampling weights were incorporated to remove the sampling bias. No missing data were found among all cortical volume measurements.

^b^ Regression coefficients measured the differences in participant structural magnetic resonance imaging variables by MTDP.

^c^ FDR correction was performed across 34 regions to prevent inflation of type I errors.

## Discussion

This cohort study found that MTDP was associated with decreased longitudinal neurocognitive development in children. These results parallel research regarding the negative effects of tobacco on neurocognitive development,^12^ indicating that tobacco can be a potent teratogen to children.

We found that children with MTDP had lower scores in the oral reading recognition and picture vocabulary tests, which measure language skills, reading recognition, and vocabulary ability.^21^ The ROI analysis showed notable differences in cortical volume for various prominent regions, such as pars orbitalis at wave 1 and the inferior parietal lobule and supramarginal gyrus at both waves. These regions are largely involved in the language process. The inferior parietal region is crucial for higher order cognition and coding of complex motor actions.^31,32^ It has a major impact with language comphresension^33,34^ and is involved with an indirect path of the arcuate fasciculus that connects to both Broca and Wernicke areas^35^ through a bundle of nerve fibers in frontal and temporal lobes of the brain.^36,37^ Wernicke area is largely involved in processing speech and language production.^38,39^ While pars orbitalis is a part of the frontal lobe, it is specifically within Broca area, which is involved in the production of speech and some language comprehension.^40^ The supramarginal gyrus plays a critical role in language development, as it is involved in the phonological processing of language.^41^ Our study also found enduring deficits in the cortical area in the precentral gyrus at both waves, consistent with a 2022 study^42^ reporting on the role of the precentral gyrus in controlling speech to ensure proper sounding of words and language fluency. The results of the NIH Toolbox Cognition Battery and the ROI analysis from structural MRI collectively demonstrate blunted development of regions involved in language skills for children with MTDP, as seen by lower crystallized cognition scores at waves 1 and 2.

In addition to deficit language skills, MTDP was associated with decreased ability regarding episodic memory as evidenced by lower scores in the picture sequence memory. Episodic memory is involved in encoding and retrieving memory regarding things in our daily experiences,^43^ and it is essential for remembering details about events or the order in which events happen.^43,44^ The ROIs with smaller volumes associated with MTDP are the posterior cingulate cortex at wave 1 and the entorhinal cortex at waves 1 and 2, and the entorhinal cortex also displayed a lower area at wave 1. The posterior cingulate cortex is crucial for memory encoding and episodic memory processing,^45,46^ and the entorhinal cortex has been noted for having a role in episodic memory processing.^47^ Our study also noted an additional region with smaller volume at both waves associated with MTDP to be the parahippocampal. Previous studies have reported that the parahippocampal gyrus is crucial in support of episodic memory.^48,49^ These regions, combined with the picture sequence memory results, demonstrate that children with MTDP may have disruptions in episodic memory.

Childhood is critical for the development of language and episodic memory. The brain continues to develop into early adulthood, with rapid growth happening during childhood.^9^ Throughout childhood, there is a progression in the growth of white matter volume, gray matter volume, and cerebral blood flow.^9^ Decreased language skills have been implicated in a host of psychological and neurological diseases.^50,51^ The results of this study suggest that MTDP is associated with blunted development of structure and function. These results are consistent with previous studies that have shown that tobacco use in utero and early childhood tobacco initiation are associated with difficulties with language processing and language skills.^12,14,15^ Our results are also consistent with previous research that has shown that exposure to tobacco in utero or with initiation in early childhood is associated with blunted neurocognitive development .^11,12,14^ Since there are lifelong implications, it is important that steps are taken to prevent MTDP.

Prevention of MTDP is critical for ensuring proper childhood brain development and language development. MTDP may have many correlations with social determinants of health. Lack of access to health care is a major factor,^52^ as communities with limited financial resources are those at highest risk of not being able to obtain health care.^52,53^ Public policy may be an effective intervention,^54^ and expansion of public support and health care access are necessary interventions.

### Limitations

This study has some limitations. First, the ABCD study lacks representation of rural communities and states in the South, Appalachia, Great Plains, and Northern Rockies.^17^ Thus, study findings might not be generalizable to these particular regions. Second, the analysis of prenatal tobacco exposure did not differentiate between exposure before or after awareness of pregnancy. Future research on cessation among those who quit after discovering pregnancy could offer valuable insights for the clinical community. Third, causal inference cannot be established based on this observational study. Prenatal exposure to tobacco may contribute to low birth weight and heighten the likelihood of irritability and hypertonicity in offspring.^55^ Future mediator studies should examine the mechanisms and pathways connecting MTDP, low birth weight, and neurocognitive development. Fourth, participants with missing data or poor neuroimaging were excluded. However, the missing rate among covariates was low, and no bias was found between complete and missing data. Fifth, the follow-up assessments for a few participants were carried out in 2020. The disruptions caused by the COVID-19 pandemic in classroom education may have impacted cognitive performance.

## Conclusions

This cohort study found that MTDP was associated with decreased cognitive function at waves 1 and 2 of the ABDCD study. These results suggest that children have poorer language processing skills and episodic memory associated with MTDP. Intervention strategies involving expanded prenatal and perinatal health availability and tobacco control policies are needed.

## Notes

Supplement 1. **eTable.** Longitudinal Comparison of Cognitive Performance among Children by MTDP using the Propensity Score Approach

Supplement 2.
Data Sharing Statement
