# Supplementary material for: Maternal Tobacco Use During Pregnancy and Child Neurocognitive Development
Source: JAMA Netw Open. 2024 Feb 13;7(2):e2355952. doi: 10.1001/jamanetworkopen.2023.55952 (PMC10865146; doi:10.1001/jamanetworkopen.2023.55952)
Supplement: Supplement 1. — eTable. Longitudinal Comparison of Cognitive Performance among Children by MTDP using the Propensity Score Approach [file jamanetwopen-e2355952-s001.pdf]

Supplemental Online Content

Puga TB, Dai HD, Wang Y, Theye E. Tobacco use during pregnancy and child neurocognitive development. *JAMA Netw Open*. 2024;7(2):e2355952. doi:10.1001/jamanetworkopen.2023.55952

eTable. Longitudinal Comparison of Cognitive Performance among Children by MTDP using the Propensity Score Approach

This supplemental material has been provided by the authors to give readers additional information about their work.

© 2024 Puga TB et al. *JAMA Network Open.*

eTable. Longitudinal Comparison of Cognitive Performance among Children by MTDP using the Propensity Score Approach[a]

NIH Toolbox Cognition Battery Weighted Mean (SE)	Wave 1			Wave 2		
	Adjusted b (SE)	Standardized b	Adjusted p-value	Adjusted b (SE)	Standardized b	Adjusted p-value
Dimensional Charge Card Sort	-1.2 (0.4)	-0.06	0.005	5.1 (2.4)	0.33	0.04
Flanker Inhibitory Control and Attention	-0.6 (0.3)	-0.03	0.04	-0.6 (0.3)	-0.04	0.06
List Sorting Working Memory	-1.9 (0.6)	-0.08	0.004	0.3 (5.7)	0.01	0.95
Oral Reading Recognition	-1.3 (0.4)	-0.10	0.003	-1.6 (0.4)	-0.12	<0.001
Pattern Comparison Process Speed	-0.5 (0.5)	-0.02	0.33	0.0 (0.5)	-0.001	0.97
Picture Sequence Memory	-2.3 (0.8)	-0.10	0.01	-2.1 (0.9)	-0.08	0.03
Picture Vocabulary Tests	-1.3 (0.5)	-0.08	0.01	-1.6 (0.5)	-0.10	0.002
Crystalized Cognition Composite Score	-1.4 (0.4)	-0.10	0.003	-1.5 (0.4)	-0.11	0.002
Fluid Cognition Composite Score	-1.8 (0.5)	-0.08	0.004	1.0 (3.6)	0.05	0.78
Total Cognition Composite Score	-1.9 (0.5)	-0.10	0.001	0.0 (2.9)	-0.0004	1.00

[a]: A propensity score (PS) model was developed to balance the covariate distribution between children with MTDP and controls (no exposure) using SAS Proc PSMATCH, where sociodemographic variables in the PS analyses include age, sex, race and ethnicity, pubertal stage, substance ever use, tobacco ever use, parental monitoring, school environment). The PS-adjusted sample weights were included in the regression models, and regression coefficients measured the difference in cognitive performance scores by MTDP.

© 2024 Puga TB et al. *JAMA Network Open.*
