# Supplementary material for: Maternal Tobacco Use During Pregnancy and Child Neurocognitive Development
Source: JAMA Netw Open. 2024 Feb 13;7(2):e2355952. doi: 10.1001/jamanetworkopen.2023.55952 (PMC10865146; doi:10.1001/jamanetworkopen.2023.55952)
Supplement: Supplement 2. — Data Sharing Statement [file jamanetwopen-e2355952-s002.pdf]

# Data Sharing Statement

Puga. Maternal Tobacco Use During Pregnancy and Child Neurocognitive Development. *JAMA Netw Open*. Published February 13, 2024. doi:10.1001/jamanetworkopen.2023.55952

**Data**
**Data available:** Yes
**Data types:** Deidentified participant data
**How to access data:** All data is publicly available through the ABCD database in the National Data Archive. https://abcdstudy.org/scientists/data-sharing/
**When available:** With publication

**Supporting Documents**
**Document types:** None

**Additional Information**
**Who can access the data:** Any data will be made available upon reasonable request.
**Types of analyses:** Any data will be made available upon reasonable request.
**Mechanisms of data availability:** Any data will be made available upon reasonable request.
**Any additional restrictions:** Any data will be made available upon reasonable request.
